# Early Surgery Does Not Improve Outcomes for Patients with Periprosthetic Femoral Fractures—Results from the Registry for Geriatric Trauma of the German Trauma Society

**DOI:** 10.3390/medicina57060517

**Published:** 2021-05-21

**Authors:** Christopher Bliemel, Katherine Rascher, Tom Knauf, Juliana Hack, Daphne Asimenia Eschbach, Rene Aigner, Ludwig Oberkircher, on behalf of the AltersTraumaRegister DGU

**Affiliations:** 1 Center for Orthopaedics and Trauma Surgery, University Hospital Giessen and Marburg, 35043 Marburg, Germany; knauft@med.uni-marburg.de (T.K.); hackj@med.uni-marburg.de (J.H.); eschbach@med.uni-marburg.de (D.A.E.); aignerr@med.uni-marburg.de (R.A.); oberkirc@med.uni-marburg.de (L.O.); 2 AUC, Akademie der Unfallchirurgie GmbH, 80639 Munich, Germany; katherine.rascher@auc-online.de

**Keywords:** periprosthetic femoral fracture, time to surgery, mortality, outcome, AltersTraumaRegister DGU^®^

## Abstract

*Background and Objectives:* Appropriate timing of surgery for periprosthetic femoral fractures (PFFs) in geriatric patients remains unclear. Data from a large international geriatric trauma register were analyzed to examine the outcome of patients with PFF with respect to the timing of surgical stabilization. *Materials and Methods:* The Registry for Geriatric Trauma of the German Trauma Society (Deutsche Gesellschaft für Unfallchirurgie (DGU)) (ATR-DGU) was analyzed. Patients treated surgically for PFF were included in this analysis. As outcome parameters, in-house mortality rate and mortality at the 120-day follow-up as well as mobility, the EQ5D index score and reoperation rate were analyzed in relation to early (<48 h) or delayed (≥48 h) surgical stabilization. *Results:* A total of 1178 datasets met the inclusion criteria; 665 fractures were treated with osteosynthesis (56.4%), and 513 fractures were treated by implant change (43.5%). In contrast to the osteosynthesis group, the group with implant changes underwent delayed surgical treatment more often. Multivariate logistic regression analysis of mortality rate (*p* = 0.310), walking ability (*p* = 0.239) and EQ5D index after seven days (*p* = 0.812) revealed no significant differences between early (<48 h) and delayed (≥48 h) surgical stabilization. These items remained insignificant at the follow-up as well. However, the odds of requiring a reoperation within 120 days were significantly higher for delayed surgical treatment (OR: 1.86; *p* = 0.003). *Conclusions:* Early surgical treatment did not lead to decreased mortality rates in the acute phase or in the midterm. Except for the rate of reoperation, all other outcome parameters remained unaffected. Nevertheless, for most patients, early surgical treatment should be the goal, so as to achieve early mobilization and avoid secondary nonsurgical complications. If early stabilization is not possible, it can be assumed that orthogeriatric co-management will help protect these patients from further harm.

## 1. Introduction

Due to an aging population in Western industrial countries, the number of performed primary and revision hip and knee arthroplasties is on the rise. Consequently, a tremendous increase in periprosthetic femoral fractures (PFFs) around the hip and knee must be expected in the future [1].

In terms of many aspects, such as patient age, comorbidities and uncertainty of gait, patients sustaining PFF are comparable to those suffering from frailty fractures of the hip and distal femur [2,3,4,5,6,7,8]. In this context, especially for patients with femoral fractures in a native hip or knee, a shortened time to surgery has been found to be beneficial, leading to reduced morbidity and mortality rates [9,10,11]. Such findings on geriatric hip and knee fractures have already been taken into account in national guidelines for several years and are furthermore used as quality indicators in the treatment of geriatric hip fracture patients [12].

Given the above-named similarities between patients with periprosthetic and native femoral fractures, it remains incomprehensible that PFF is still often affiliated with a considerable delay in time to surgery [13,14]. Rather, it has to be presumed that if there would be sufficient evidence of an early surgical treatment to be beneficial for these patients as well, often attempted explanations, such as the complexity of the surgical procedure or the provision of a magnitude of appropriate implants, would be finally addressed.

Currently, the literature on this topic remains limited and contradictory. While some studies report on the positive effects of early surgical fixation of PFF [13,15], other studies found no or only partially beneficial effects on short- to mid-term perioperative outcomes and mortality in PFF patients [16,17].

To provide more clarity concerning this controversial point of debate, the purpose of the present study was to analyze the data of the Registry for Geriatric Trauma (AltersTraumaRegister DGU^®^ (ATR-DGU)) of the German Trauma Society (Deutsche Gesellschaft für Unfallchirurgie (DGU)).

It was hypothesized that compared to delayed surgical treatment (≥48 h), early surgical treatment (<48 h) would lead to decreased rates of perioperative complications and mortality among PFF patients.

## 2. Materials and Methods

### 2.1. ATR-DGU

The source of the data in the present analysis is ATR-DGU (http://www.alterstraumaregister-dgu.de, accessed on 9 April 2021). The ATR-DGU is a large prospective, multicenter, standardized and anonymous registry founded by the German Trauma Society. ATR-DGU provides information on geriatric trauma patients with hip, periprosthetic and peri-implant femoral fractures.

The infrastructure for documentation, data management and data analysis is provided by the Academy for Trauma Surgery (AUC—Akademie der Unfallchirurgie GmbH). Scientific management was carried out by the Working Committee on Geriatric Trauma Registry (AK ATR) of the German Trauma Society (DGU). Every certified Center for Geriatric Trauma (AltersTraumaZentrum DGU^®^) is obliged to participate in the ATR-DGU. Participating centers transmit pseudonymized patient data via a web-based application into a central database. The standard documentation sheet contains approximately 160 data fields per patient. Currently, hospitals from three countries (Germany, Switzerland and Austria) contribute to the ATR-DGU, with a total of almost 25,000 cases from about 100 hospitals. Data reported in this study were collected preoperatively in the years 2016 to 2019, at the time of patient’s hospital admission, during surgery, one week after surgery and optionally at 120 days postoperatively. The ATR-DGU contains detailed information on the demographics, preoperative residential and health status, comorbidities, fracture pattern, time course, relevant medication history and outcome of each individual. Approval for scientific data analysis from the ATR-DGU is granted via a peer-review process in accordance with the publication guidelines laid down by the AK ATR. The present study is in accordance with the publication guidelines of the ATR-DGU and registered as ATR-DGU project ID 2020-003.

### 2.2. Aim of the Study and Outcome Parameters

**Figure 1:**
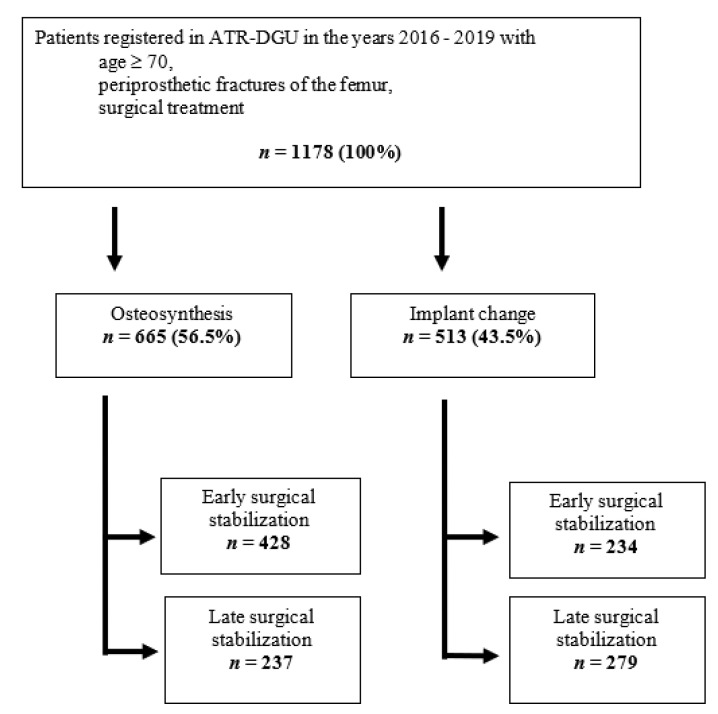
Flow sheet of the included population.

The aim of the study was to analyze the differences in mortality rate during the stay in an acute care hospital and at the 120-day follow-up, depending on the time to surgery. In accordance with current research, early surgical stabilization was defined as surgical treatment performed within the first 48 h after hospital admission, whereas late stabilization was defined as surgical treatment after 48 h [16,18,19,20]. Univariable outcomes were examined separately for patients who received an implant exchange and those who received an osteosynthesis (Figure 1). Other outcomes studied were the mobility of patients and the EQ-5D at seven and 120 days after the surgical treatment as well as the mobility and reoperation rate at 120 days following the surgical treatment.

The present analysis covered the following data: American Society of Anesthesiologists (ASA) score, time to surgery, Identification of Seniors At Risk (ISAR) score [21], age, sex, residential status, concomitant fractures, walking ability (before the fracture, on the seventh day after surgery and at 120 days follow-up), length of hospital stay, type of surgery, surgical complication (at the initial stay and at 120 days follow-up), discharge after hospital and current location at the follow-up, EQ-5D (on the seventh day after surgery and follow-up), mortality (initial stay and at 120 days follow-up) and anticoagulation on admission. As the focus of the present analysis was time to surgery, only anticoagulatory drugs that led to a delay in surgical treatment were considered anticoagulatory medications (e.g., vitamin K antagonists, selective thrombin inhibitors and selective direct Xa inhibitors). Other anticoagulatory medications, such as acetylsalicylic acid, other antiplatelet drugs (e.g., clopidogrel) and heparinoids that normally do not result in a delay in surgical treatment, were not considered anticoagulatory drugs for this analysis.

### 2.3. Inclusion and Exclusion Criteria

Patients are only included in the ATR-DGU if they are 70 years or older and have undergone surgery for a hip fracture and periprosthetic or peri-implant fractures of the femur. This study focused solely on PFF due to inserted total hip arthroplasty (THA) or total knee arthroplasty (TKA) (*n* = 1193 patients). Pertrochanteric femoral fractures as well as femoral neck fractures and peri-implant femoral fractures were excluded. Only datasets where the time to surgery was documented were used for this analysis (*n* = 1178 patients from 85 hospitals).

### 2.4. Statistical Analysis

All calculations were performed via the statistics software R v. 4.0.2 (Foundation for Statistical Computing, Vienna, Austria). For descriptive analyses, categorical data are presented as counts and percentages, and continuous variables are presented as medians with interquartile ranges (IQRs). The results of the EQ-5D-3 L questionnaires were transformed to a single value using the time trade off algorithm validated for Germany. This value ranges from 0 for the worst health status to 1 for the best health status. Some patients had missing data for individual parameters; therefore, each analysis shows the total number of patients who were analyzed. Comparisons between groups (early vs. delayed time to surgery) were made using Χ^2^-test for categorical variables and the Mann–Whitney U test for continuous variables. Linear and logistic regression models were used to examine the impact of early vs. late time to surgery on a range of outcomes (mortality, mobility of patients, quality of life and reoperation rates) after controlling for sex, ASA score, anticoagulatory medication at admission and implant change. The results are reported as regression coefficients (ß) for linear regression and odds ratios (ORs) for logistic regression along with their 95% confidence intervals (CIs). Differences were considered statistically significant when *p* < 0.05.

## 3. Results

### 3.1. Acute Care Data

A total of 1178 datasets from geriatric trauma patients with PFF met the inclusion criteria. Analysis in terms of the treatment option (osteosynthesis or implant exchange) revealed that a slight majority of fractures were treated with osteosynthesis (*n* = 665; 56.4%), whereas *n* = 513 fractures (43.5%) were treated by implant replacement (Figure 1). A tabular presentation of the patient data included in this study, depending on the kind of surgical treatment, is illustrated in Table 1. Analysis of patient’s age revealed that patients suffering from PFF were predominantly between their late 70s and 80s of age, with values of the 25th quartile ranging at 79 years for both treatment groups. The value of the 75th quartile was at 88 years for osteosynthetic fixation and, therefore, slightly extended as compared to those receiving prosthetic treatment (87 years) (Table 1).

Whereas early surgical stabilization was more frequently performed in the osteosynthesis group, delayed surgical treatment was predominantly carried out in the group with implant changes (Table 2). Values of the ASA score were comparable for patients treated with osteosynthesis and implant change (*p* = 0.393 and *p* = 0.212) regardless of the time of supply, with approximately 80% of patients being estimated as ASA ≥3 in both groups. Values of ISAR score showed the majority of patients to be ISAR ≥2 (88.4% for osteosynthesis and 86.2% implant change), indicating the need for an increased geriatric action (*p* = 0.129 and *p* = 0.102).

Prior to the fracture, patients predominantly lived at home (73.9% for osteosynthesis and 79.5% implant change), with the majority of them reporting a walking inability or at least a certain unsteadiness of gait, as indicated by the use of walking sticks, crutches or walkers, before the fracture (64.7% for osteosynthesis (*p* = 0.412) and 58.9% implant replacement (*p* = 0.189)). These difficulties in patient mobility became even more serious seven days after the surgical treatment. At this point of time, almost 100% of patients relied on aids for mobilization or were immobile. Ultimately, this resulted in the fact that more than 20% of all patients could be discharged straight home (*p* = 0.795 and *p* = 0.053) (Table 2).

After controlling for sex, age, ASA score, anticoagulation and implant exchange, no difference could be found between the early and delayed surgical groups in terms of mortality during the initial hospital stay (*p* = 0.310). Walking ability at seven days post-surgery (*p* = 0.239) and quality of life seven days post-surgery as measured by the EQ5D index (*p* = 0.812) also showed no significant difference in either group (Table 3).

### 3.2. 120 Days Follow-Up Data

For 569 geriatric trauma patients, data were available for the 120-day follow-up (Table 4). The rate of readmission to the hospital within these first 120 days ranged between 5.8 and 7.7% for patients treated with osteosynthesis and between 10.1 and 12.9% for patients with implant changes dependent on early or delayed surgical treatment, without statistical significance (*p* = 0.727 and *p* = 0.702). A similar picture emerged for the rate of reoperations within the first 120 days after the initial surgery. Additionally, at this item, no significant difference related to time to surgery could be found for osteosynthetic (*p* = 0.320) and prosthetic (*p* = 0.538) treatment of PFF.

The ability to walk independently or to walk outside with walking aids was also comparable between early or late osteosynthetic surgical stabilization (*p* = 0.449) and implant replacement (*p* = 0.498) (Table 4). In terms of residency 120 days following surgery, no differences related to time to surgery were observed in the osteosynthesis group (*p* = 0.996) and the implant change group (*p* = 0.605) (Table 4). Additionally, the mortality rate remained unaffected by the time point of surgical stabilization (osteosynthesis *p* = 0.996 and implant exchange *p* = 1.000).

Multivariate analysis of parameters that had increased at the follow-up showed that the odds ratio of reoperation within 120 days postoperatively was significantly higher in the delayed intervention group (OR: 1.86; *p* = 0.003, Table 3). However, 120 days post-surgery mortality rate, patients’ walking ability and quality of life as measured by the EQ5D index score did not differ between early and delayed surgical treatment (Table 3).

## 4. Discussion

This register study aimed to analyze the impact of time to surgery on patients with PFF. The principal findings revealed that surgical treatment within the first 48 h following hospital admission did not lead to decreased mortality rates either during the stay in an acute hospital or within the first 120 days of follow-up. Walking abilities, such as discharge management and residential status at the time of the 120-day follow-up, remained unaffected by delayed surgical stabilization. Nevertheless, multivariate regression analysis showed a statistically increased rate of reoperation when fracture fixation was performed after a 48 h cutoff.

The results of this present analysis are therefore in line with those of Bovonratwet et al., who reported on patients suffering exclusively from periprosthetic hip fractures with a follow-up of 30 days [16]. Their study also dichotomized the time to surgery at 48 h. Following the statements of Bovonratwet et al., neither mortality nor a variety of different perioperative complications (e.g., urinary tract infection, wound complication, pneumonia) were influenced by a delay in time to surgery [16]. Similar results were published by Lee and coworkers, who reported 263 patients with TKA suffering from PFF [20]. Their analysis focused on rates of intra- and postoperative complications dependent on early or delayed surgical treatment. Additionally, in their patient population the different types of complications remained unaffected by time to surgery at a 48 h cutoff [20]. Nevertheless, Bhattacharyya et al. reported inferior outcomes following delayed surgical treatment in patients with PFF and dichotomization at 48 h [18]. Having retrospectively analyzed the clinical courses of 106 patients suffering from PFF, they found a 1.27 times higher probability of dying within the first year following a fracture if patients had a delay of surgery of more than two days. Hence, these results are contrary to our findings and the findings of the above-named research contributions. A possible explanation for this might be the difference in the length of follow-up. While Bhattacharyya et al. report on data with a one-year follow-up, ATR-DGU only reports acute care data, such as 120-day follow-up data. Nevertheless, mentioning the retrospective analysis from Sellan et al., reporting 180 patients with PFF (111 THA, 69 TKA), it has to be doubted that an extension of the follow-up period would have affected our own results. In their study, Sellan et al. concluded that both the postoperative length of hospital stay and mortality rate one year after surgical treatment remained unaffected by a time to surgery greater than 48 h [19].

Other studies focusing on mortality in patients with PFF suggested different time intervals to separate early from delayed surgical treatment, e.g., 24 h [17,22,23] or 72 h [15,24]. A separate analysis of ATR-DGU using 24 and 72 h as cutoffs also failed to prove differences in regard to mortality (data not shown).

It is worth noting that this lack of relationship between mortality rate and delay to surgery represents not the only aspect demonstrating that PPF is probably not a typical frailty marker. Other than hip fracture patients [25,26], in addition to mortality and patients’ walking abilities seven days after surgical treatment, discharge management from the hospital and the patient’s residential status at the time of the 120-day follow-up remained unaffected by a delay in surgical stabilization. In regard to parameters determined during the stay at the acute hospital, it is worth noting that all patients included in this analysis were treated in certified orthogeriatric trauma centers. These centers provide access to orthogeriatric co-management under the best possible conditions that might also cushion the presumed negative effects of delayed surgical treatment in patients with such a fracture pattern.

Nevertheless, the significantly increased rate of reoperations, determined for patients with a delay in surgical treatment at the 120-day follow-up interview, is unlikely to be affected by the benefits of orthogeriatric co-management, as this issue predominantly represents a surgical problem. A closer look at the types of complications revealed that wound lavage of seromas and hematomas were predominantly represented in the groups with delayed surgical treatment. However, the most obvious cause of such complications, the use of anticoagulants, is invalidated due to its consideration as a possible confounder in the multivariate regression analysis. Furthermore, it is noticeable that among patients with delayed surgical treatment an increased number of individuals underwent conversion to arthroplasty as a secondary intervention. Unfortunately, an exact analysis of this effect cannot be carried out due to the register data. However, it must be noted that these results are contrary to the data previously published in the literature [16,17,23]. A possible explanation for such discordant findings may be due to different baseline patient characteristics, including differences in age, sex and medical comorbidity burden, as well as differing durations of follow-up.

### Limitations

Because the present analysis is based on registry data, some limitations must be recognized. Despite similar demographic data and comorbidities of patients listed in the early and late surgical groups, the possibility cannot be ruled out that attending physicians treated more frail patients later in the clinical course. Additionally, it must be noted that PPFs are quite heterogeneous in terms of the surgical challenge they pose to the treating surgeons. Delayed fracture stabilization after the first 48 h can also be interpreted as indirect evidence of the complexity of the fracture. Regarding the latter, two main reasons are conceivable, especially in revision arthroplasty. First, only a small number of surgeons have the required expertise to carry out such operations; second, many hospitals do not have direct access to revision arthroplasty sets, often requiring ordering, causing further delays. Other in osteosynthetic fracture fixation, such implants are routinely more available and a much larger number of orthopedic surgeons have surgical expertise with the procedure. While well-designed randomized trials can prove causality, registry analyses such as this one can only describe associations. Our findings must therefore be interpreted with caution. These findings should be further tempered by the fact that there is a certain heterogeneity of the patient population included, with PFF at an underlying hip and knee arthroplasty. Due to limitations of the standard documentation sheet so far, it remains unknown how many of the included PFFs were exactly caused by fractures at a hip prosthesis or a knee prosthesis. A recent revision of the standard documentation sheet should allow a more precise statement on this issue in the future. Additionally, a lack in important patients’ data such as the body mass index, mean time from the primary fracture to the periprosthetic femoral fracture and biochemical values of protein C and erythrocyte sedimentation that rule out infectious processes has to be mentioned, which further restricts the results of this register analysis. Finally, it must be noted that the assumed positive influence of orthogeriatric treatment on outcomes in patients with PFF cannot be conclusively assessed with the present analysis due to the absence of a control group. Further studies are therefore needed to evaluate the efficacy of orthogeriatric co-management in patients with PFF, as it has already been proven to be beneficial for other geriatric fractures [27]. Finally, it should be noted that the data presented within this present study represent one of the largest prospectively collected collectives of periprosthetic femoral fractures so far, which definitively strengthens the findings obtained.

## 5. Conclusions

The results of the present register analysis further support current research, as they revealed that early surgical treatment of patients with PFF did not lead to decreased mortality rates in the acute phase or in the midterm. Except for the rate of reoperation, all other outcome parameters remained unaffected. Nevertheless, for most patients early surgical treatment should be the goal, so as to achieve early mobilization and avoid nonsurgical secondary complications. If early stabilization for any reason is not possible, it can be presumed that a profound preoperative evaluation and preparation under orthogeriatric co-management is appropriate for protecting patients from further harm.

## Figures and Tables

**Table 1:** Data on geriatric trauma patients with periprosthetic femoral fractures dependent on the kind of treatment.

Parameter	Subdivision	Osteosynthesis	Implant Change	*p*-Value
**Number of patients**		665	513	
**Gender**	Male	147 (22.3%)	159 (31.0%)	0.001 *
	Female	513 (77.7%)	354 (69.0%)	
**Patient age (year)** **Median**		84 (79; 88)	83 (79; 87)	0.002 **
**Time to surgery (h)** **Median**		27.0 (13.7; 69.7)	57.1 (24.6; 106)	<0.001 **
**ASA score**	Unknown	15	17	0.678 *
	1	4 (0.6%)	4 (0.8%)	
	2	130 (20.0%)	109 (22.1%)	
	3	453 (69.8%)	331 (67.1%)	
	4 and 5	62 (9.6%)	49 (9.9%)	
**ISAR score**	0	32 (7.5%)	24 (7.0%)	0.012 *
	1	45 (10.6%)	47 (13.6%)	
	2	102 (24%)	87 (25.2%)	
	3	85 (20%)	96 (27.8%)	
	4	109 (25.6%)	61 (17.7%)	
	5	45 (10.6%)	22 (6.4%)	
	6	7 (1.7%)	8 (2.3%)	
**Anticoagulatory drugs**	Yes	153 (23.3%)	114 (22.6%)	0.832 *
	No	503 (76.7%)	390 (77.4%)	
**Prefracture** **residental status**	At home	492 (75.8%)	408 (81.4%)	0.040 *
	Nursing home	139 (21.4%)	74 (14.8%)	
	Hospital	12 (1.9%)	14 (2.8%)	
	Other	6 (0.9%)	5 (1.0%)	
**Cocomitant fractures**	Yes	48 (7.3%)	41 (8.1%)	0.714 *
	No	609 (92.7%)	468 (91.9%)	
**Prefracture walking ability**	Unknown	47	46	0.024 *
	Independent without walking aids	181 (29.6%)	165 (35.5%)	
	Ability to walk outside with a walking stick or crutch	100 (16.4%)	80 (17.1%)	
	Ability to walk outside with two crutches or a walker	213 (34.9%)	148 (31.7%)	
	Certain walking ability in the apartment, but outside only with an assistant	79 (12.9%)	62 (13.3%)	
	No functional walking ability	38 (6.2%)	12 (2.6%)	
**Death during stay in the acute hospital**	Yes	21 (3.2%)	21 (4.1%)	0.500
	No	639 (96.8%)	492 (95.9%)	
**Ability to walk at the 7th postoperative day**	Unknown	15	18	0.031 *
	Without aid	3 (0.5%)	0 (0%)	
	With walking stick or crutch	51 (7.9%)	46 (9.3%)	
	With a rollator	89 (13.8%)	90 (18.3%)	
	With a goat	86 (13.3%)	62 (12.6%)	
	With a walker	144 (22.3%)	125 (25.4%)	
	Not possible	274 (42.3%)	170 (34.5%)	
**Reoperation within initial acute hospital stay** (several surgical procedures per patient possible)	Reposition	1 (3.3%)	6 (12.5%)	0.024 *
	Lavage/debridement	10 (33.3%)	22 (45.8%)	
	Implant removal	1 (3.3%)	3 (6.3%)	
	Periprosthetic fracture	4 (13.3%)	1 (2.1%)	
	Other	14 (46.7%)	16 (33.3%)	
**Discharge from hospital**	At home	153 (23.4%)	108 (21.2%)	0.001 *
	Nursing home	238 (36.3%)	136 (26.7%)	
	Inpatient stay/other	243 (37.1%)	245 (48.0%)	
	Death	21 (3.2%)	21 (4.1%)	

* Chi-square test; ** Mann–Whitney test.

**Table 2:** Data on geriatric trauma patients with periprosthetic femoral fractures dependent on time to surgery and kind of treatment.

Parameter	Subdivision	Osteosynthesis			Implant Change		
		Early Surgical Stabilisation	Late Surgical Stabilisation	*p*-Value	Early Surgical Stabilisation	Late Surgical Stabilisation	*p*-Value
**Number of patients**		428 (75.8%)	237 (24.2%)		234 (46.6%)	279 (54.4%)	
**Gender**	Male	76 (18.0%)	71 (30.0%)	0.001 *	70 (29.9%)	89 (31.9%)	0.698 *
	Female	347 (82.0%)	166 (70.0%)		164 (70.1%)	190 (68.1%)	
**Patient age (year)** **Median**		85(80; 89)	84 (79; 87)	0.027 **	83 (79; 87)	83 (79; 87)	0.663 **
**Time to surgery (h)** **Median**		18.6	89.6		24	103	
**ASA score**	Unknown	13	2	0.393 *	4	13	0.212 *
	1	2 (0.4%)	2 (0.9%)		0 (0.0%)	4 (1.51%)	
	2	89 (21.4%)	41 (17.5%)		54 (23.7%)	55 (20.8%)	
	3	281 (67.7%)	172 (73.5%)		154 (67.5%)	177 (66.8%)	
	4 and 5	43 (10.4%)	19 (8.1%)		20 (8.77%)	29 (10.9%)	
**ISAR score**	0	25 (9.3%)	7 (4.5%)	0.129 *	15 (9.4%)	9 (4.9%)	0.102 *
	1	30 (11.2%)	15 (9.6%)		25 (15.6%)	22 (11.9%)	
	2	57 (21.2%)	45 (28.8%)		38 (23.8%)	49 (26.5%)	
	3	50 (18.6%)	35 (22.4%)		38 (23.8%)	58 (31.4%)	
	4	98 (25.3%)	41 (26.3%)		33 (20.6%)	28 (15.1%)	
	5	33 (12.3%)	12 (7.7%)		10 (6.3%)	12 (6.5%)	
	6	6 (2.2%)	1 (0.6%)		1 (0.6%)	7 (3.8%)	
**Anticoagulatory drugs**	Yes	90 (21.2%)	63 (27.3%)	0.096 *	43 (18.8%)	71 (25.8%)	0.076 *
	No	335 (78.8%)	168 (72.7%)		186 (81.2%)	204 (74.2%)	
**Prefracture** **residental status**	At home	310 (74.7%)	182 (77.8%)	0.280 *	188 (81.7%)	220 (81.2%)	0.802 *
	Nursing home	95 (22.9%)	44 (18.8%)		32 (13.9%)	42 (15.5%)	
	Hospital	8 (1.9%)	4 (1.7%)		8 (3.5%)	6 (2.2%)	
	Other	2 (0.5%)	4 (1.7%)		2 (0.9%)	3 (1.1%)	
**Cocomitant fractures**	Yes	27 (6.4%)	21 (8.9%)	0.298 *	18 (7.7%)	23 (8.3%)	0.930 *
	No	395 (93.6%)	214 (91.1%)		215 (92.3%)	253 (91.7%)	
**Prefracture** **walking ability**	Unknown	36	11	0.412 *	24	22	0.189 *
	Independent without walking aids	113 (29.2%)	68 (30.4%)		82 (39.0%)	83 (32.3%)	
	Ability to walk outside with a walking stick or crutch	59 (15.2%)	41 (18.3%)		37 (17.6%)	43 (16.7%)	
	Ability to walk outside with two crutches or a walker	132 (34.1%)	81 (36.2%)		64 (30.5%)	84 (32.7%)	
	Certain walking ability in the apartment, but outside only with an assistant	56 (14.5%)	23 (10.3%)		25 (11.9%)	37 (14.4%)	
	No functional walking ability	27 (7.0%)	11 (4.9%)		2 (1.0%)	10 (3.9%)	
**Death during stay in the acute hospital**	Yes	13 (3.1%)	8 (3.4%)	1.000 *	13 (5.5%)	8 (2.9%)	0.191 *
	No	411 (96.9%)	228 (96.6%)		221 (94.4%)	271 (97.1%)	
**Ability to walk at the 7th postoperative day**	Unknown	11	4	<0.001 *	6	12	0.167 *
	Without aid	0	3 (1.3%)		0 (0%)	0 (0%)	
	With walking stick or crutch	34 (8.2%)	17 (7.3%)		25 (11.1%)	21 (7.9%)	
	With a rollator	56 (13.5%)	33 (14.2%)		39 (17.3%)	51 (19.1%)	
	With a goat	198 (47.8%)	76 (32.6%)		67 (29.6%)	103 (38.6%)	
	With a walker	49 (11.8%)	37 (15.9%)		33 (14.6%)	29 (10.9%)	
	Not possible	77 (18.6%)	67 (28.8%)		62 (27.4%)	63 (23.6%)	
**Reoperation within initial acute hospital stay** (several surgical procedures per patient possible)	Reposition	1 (4.4%)	0 (0%)	0.150 *	2 (11.8%)	4 (12.9%)	0.212 *
	Lavage/debridement	8 (34.8%)	2 (28.6%)		7 (41.2%)	15 (48.4%)	
	Implant removal	0 (0%)	1 (14.3%)		2 (11.8%)	1 (3.2%)	
	Periprosthetic fracture	3 (13.0%)	1 (14.3%)		0 (0.0%)	1 (3.2%)	
	Other	11 (47.8%)	3 (42.9%)		6 (35.3%)	10 (32.3%)	
**Discharge from hospital**	At home	101 (23.9%)	52 (22.3%)	0.795 *	57 (24.6%)	51 (18.3%)	0.053 *
	Nursing home	157 (37.2%)	81 (34.8%)		64 (27.6%)	72 (25.9%)	
	Inpatient stay/other	151 (35.8%)	92 (39.5%)		98 (42.2%)	147 (52.9%)	
	Death	13 (3.1%)	8 (3.4%)		13 (5.6%)	8 (2.9%)	

* Chi-square test; ** Mann–Whitney test.

**Table 3:** Multivariate logistic and linear regression analysis of time to surgery <48 h vs. ≥48 h (reference group). Analysis is adjusted for sex, patient age, ASA score, anticoagulation and implant replacement.

**Influence of Time to Surgery on…**	***N***		**OR**	**95% CI and OR**	***p*-Value**
**Acute phase**					
	**<48 h**	**≥48 h**			
**Death during stay in the acute hospital** * Yes vs. no	642	504	0.70	(0.35; 1.38)	0.310
**Walking ability after 7 days** * Unable to walk, e.g., just in the room/flat vs. able to walk	624	489	0.86	(0.66; 1.11)	0.239
	***N***		**ẞ**	**95% CI and ẞ**	***p*-Value**
**EQ5D index after 7 days** ~	446	351	−0.005	(−0.05; 0.04)	0.812
**120 days follow-up**					
	**<48 h**	**≥48 h**			
**Mortality** *	258	144	0.92	(0.49; 1.70)	0.803
**Walking ability** * Unable to walk, e.g., just in the room/flat vs. able to walk	232	128	0.77	(0.47; 1.27)	0.307
**Reoperation within the first 120 days postoperative**	318	215	1.86	(1.23; 2.83)	0.003
	***N***		**ẞ**	**95% CI and ẞ**	***p*-Value**
**EQ5D index** ~	191	101	−0.07	(−0.14; 0.01)	0.082

* Logistic regression; ~ linear regression.

**Table 4:** 120-day follow-up data on geriatric trauma patients with periprosthetic femoral fractures dependent on time to surgery and kind of treatment.

Parameter	Subdivision	Osteosynthesis			Implant Change		
		Early Surgical Stabilisation	Late Surgical Stabilisation	*p*-Value	Early Surgical Stabilisation	Late Surgical Stabilisation	*p*-Value
**Number of patients**		226 (67.5%)	109 (32.5%)		114 (48.7%)	120 (51.3%)	
**Readmission to hospital**	Yes	11 (5.8%)	7 (7.7%)	0.727 *	10 (10.1%)	12 (12.9%)	0.702 *
	No	179 (94.2%)	84 (92.3%)		89 (89.9%)	81 (87.1%)	
**Reoperation within the first 120 days** **postoperative** (several surgical procedures per patient possible)	Reposition	0 (0%)	0 (0%)	0.320 *	6 (60%)	3 (37.5%)	0.538 *
	Lavage/debridement	0 (0%)	3 (37.5%)		2 (20%)	3 (37.5%)	
	Implant removal	0 (0%)	2 (25%)		1 (10%)	0 (0%)	
	Conversion in total arthroplasty	0 (0%)	2 (25%)		0 (0%)	1 (12.5%)	
	Periprosthetic fracture	5 (55.6%)	1 (12.5%)		0 (0%)	0 (0%)	
	Other	4 (44.4%)	0 (0%)		1 (10%)	1 (12.5%)	
**Walking ability at the time of the 120 days follow-up**	Unknown	59	38	0.449 *	29	52	0.498 **
	Independent without walking aids	8 (5.1%)	4 (6.0%)		6 (7.2%)	2 (3.1%)	
	Ability to walk outside with a walking stick or crutch	22 (14.1%)	7 (10.4%)		16 (19.3%)	10 (15.4%)	
	Ability to walk outside with two crutches or a walker	67 (42.9%)	34 (50.7%)		32 (38.6%)	32 (49.2%)	
	Certain walking ability in the apartment, but outside only with an assistant	29 (18.6%)	15 (22.4%)		12 (14.5%)	6 (9.2%)	
	No functional walking ability	30 (19.2%)	7 (10.4%)		17 (20.5%)	15 (23.1%)	
**Residence at the time of the 120 days follow-up**	At home	92 (52%)	39 (53.4%)	0.996	60 (65.9%)	47 (63.5%)	0.605 *
	Nursing home	52 (29.4%)	21 (28.8%)		16 (17.6%)	12 (16.2%)	
	Inpatient stay/other	7 (4.0%)	3 (4.1%)		3 (3.3%)	6 (8.1%)	
	Death	26 (14.7%)	10 (13.7%)		12 (13.2%)	9 (12.2%)	
**Death within the first 120 days postoperative**	Yes	26 (14.7%)	10 (13.7%)	0.996 *	12 (13.2%)	9 (12.2%)	1000 *
	No	151 (85.3%)	63 (86.3%)		79 (86.8%)	65 (87.8%)	

* Chi-square test; ** Mann–Whitney test.

## Data Availability

The analyzed datasets of this study are available from the corresponding author on reasonable request.
